# Recent advances in porous nanomaterials-based drug delivery systems for cancer immunotherapy

**DOI:** 10.1186/s12951-022-01489-4

**Published:** 2022-06-14

**Authors:** Su-Ran Li, Fang-Yi Huo, Han-Qi Wang, Jing Wang, Chun Xu, Bing Liu, Lin-Lin Bu

**Affiliations:** 1 The State Key Laboratory Breeding Base of Basic Science of Stomatology (Hubei-MOST) & Key Laboratory of Oral Biomedicine Ministry of Education, School and Hospital of Stomatology, Wuhan University, Wuhan, 430072 China; 2 School of Dentistry, The University of Queensland, Herston, QLD 4006 Australia; 3 Department of Oral Maxillofacial Head Neck Oncology, School and Hospital of Stomatology, Wuhan University, Wuhan, 430079 Hubei China

**Keywords:** Porous nanomaterials, Drug delivery systems, Inorganic porous nanomaterials, Metal–organic framework (MOFs), Cancer immunotherapy

## Abstract

Cancer immunotherapy is a novel therapeutic regimen because of the specificity and durability of immune modulations to treat cancers. Current cancer immunotherapy is limited by some barriers such as poor response rate, low tumor specificity and systemic toxicities. Porous nanomaterials (PNMs) possess high loading capacity and tunable porosity, receiving intense attention in cancer immunotherapy. Recently, novel PNMs based drug delivery systems have been employed in antitumor immunotherapy to enhance tissue or organ targeting and reduce immune-related adverse events. Herein, we summarize the recent progress of PNMs including inorganic, organic, and organic–inorganic hybrid ones for cancer immunotherapy. The design of PNMs and their performance in cancer immunotherapy are discussed in detail, with a focus on how those designs can address the challenges in current conventional immunotherapy. Lastly, we present future directions of PNMs for cancer immunotherapy including the challenges and research gaps, providing new insights about the design of PNMs for efficient cancer immunotherapy with better performance as powerful weapons against tumors. Finally, we discussed the relevant challenges that urgently need to be addressed in clinical practice, coupled with corresponding solutions to these problems.

## Introduction

**Fig. 1:**
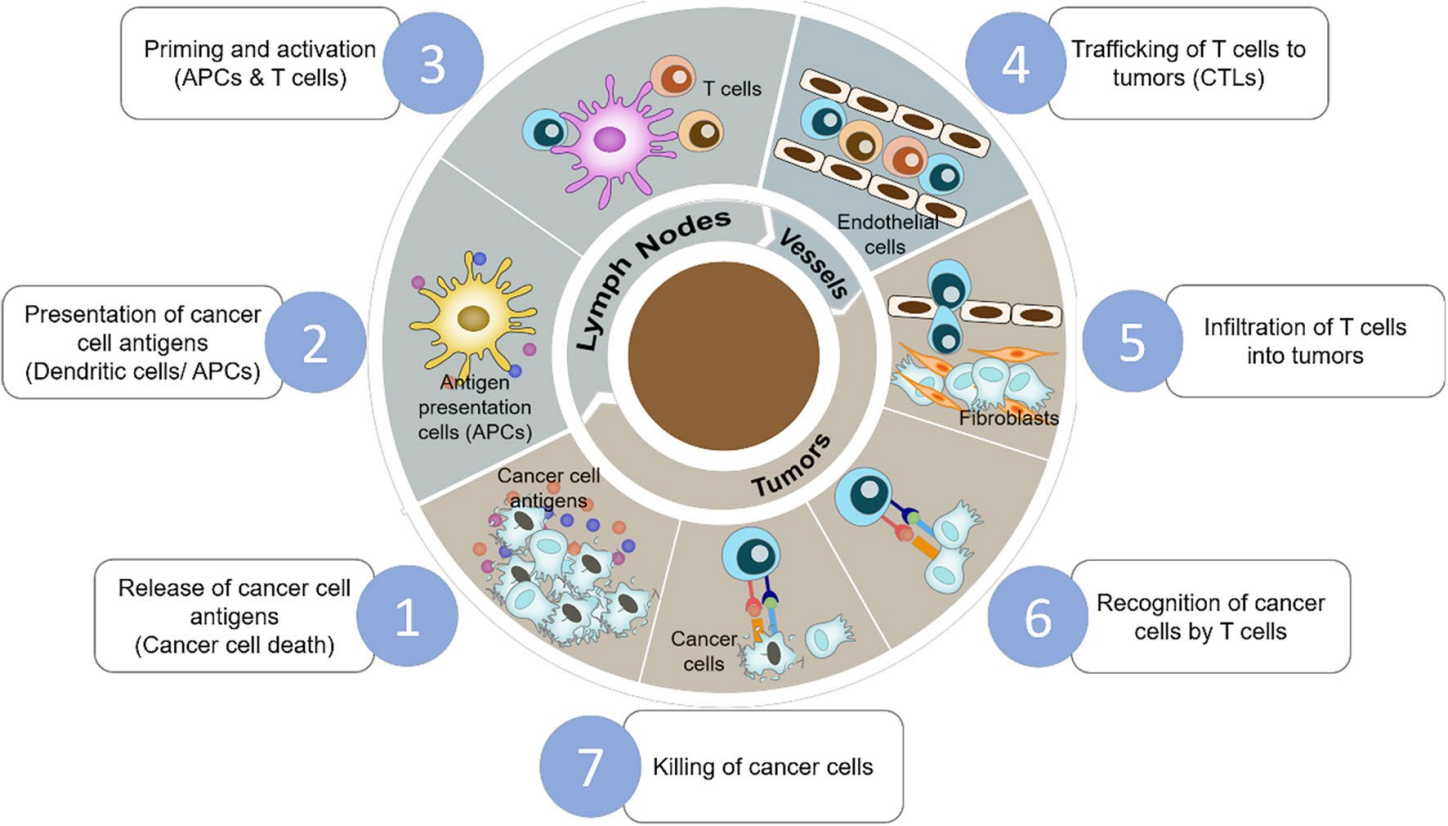
The immuno-oncology cycle. This cycle is comprised of seven parts, including the release of cancer cell antigens, antigen presentation by mature antigen presenting cells (APCs), T cell activation, T cell trafficking and infiltration into tumor sites, and the recognition and killing of cancer cells by cytotoxic T cells (CTLs). (Adapted from [127])

Cancer is one of the leading causes of death and brings an increasing socioeconomic burden worldwide [1, 2]. Recently, immunotherapy has become a powerful and innovative clinical option for treating cancers owing to its capacity for long-lasting responses and tissue targeting ability [3]. Under healthy conditions, the immune system can eliminate tumor cells efficiently by self-sustaining and self-restricting feedback loops through the cancer-immunity cycle (Fig. 1). However, tumors develop strategies to evade immune surveillance and impair the anti-tumor immune response in patients with cancer [4, 5]. Currently, cancer immunotherapy manipulates the immune system from three main aspects: (1) immune checkpoint blockade (ICB) therapy, which blocks checkpoint proteins such as programmed death protein 1 (PD-1), programmed death-ligand 1(PD-L1), cytotoxic T-lymphocyte-associated protein 4 (CTLA-4) to allow T cells to kill cancer cells; (2) cancer vaccines and (3) adoptive-cell-transfer (ACT) therapy [6–8]. Cancer immunotherapy elicits powerful immune responses to treat primary tumors and inhibits their metastasis and relapse [9]. Cancer immunotherapy can avoid multiple drug resistance, reduce genetic mutation in tumor cells, and augment synergistic therapeutic effects with other treatments, such as chemotherapy, radiotherapy, photodynamic therapy (PDT), and photothermal therapy (PTT) [10, 11].

Despite these favorable features and some promising clinical outcomes, there are still some challenges for cancer immunotherapy. For example, direct administration of ICB antibodies may have off-target toxicity due to lacking cancer cell specificity [12]. For cancer vaccines, inefficient uptake and presentation by antigen-presentation cells (APCs) [13, 14] may result in insufficient immune responses. Those limitations may come from lacking efficient methods to deliver those therapeutic agents to the target place. Additionally, the therapeutic efficacy of cancer immunotherapy is largely limited by the immunosuppressive tumor microenvironment [15]. These barriers to effective cancer immunotherapy need to be addressed for better future clinical efficacy.

**Fig. 2:**
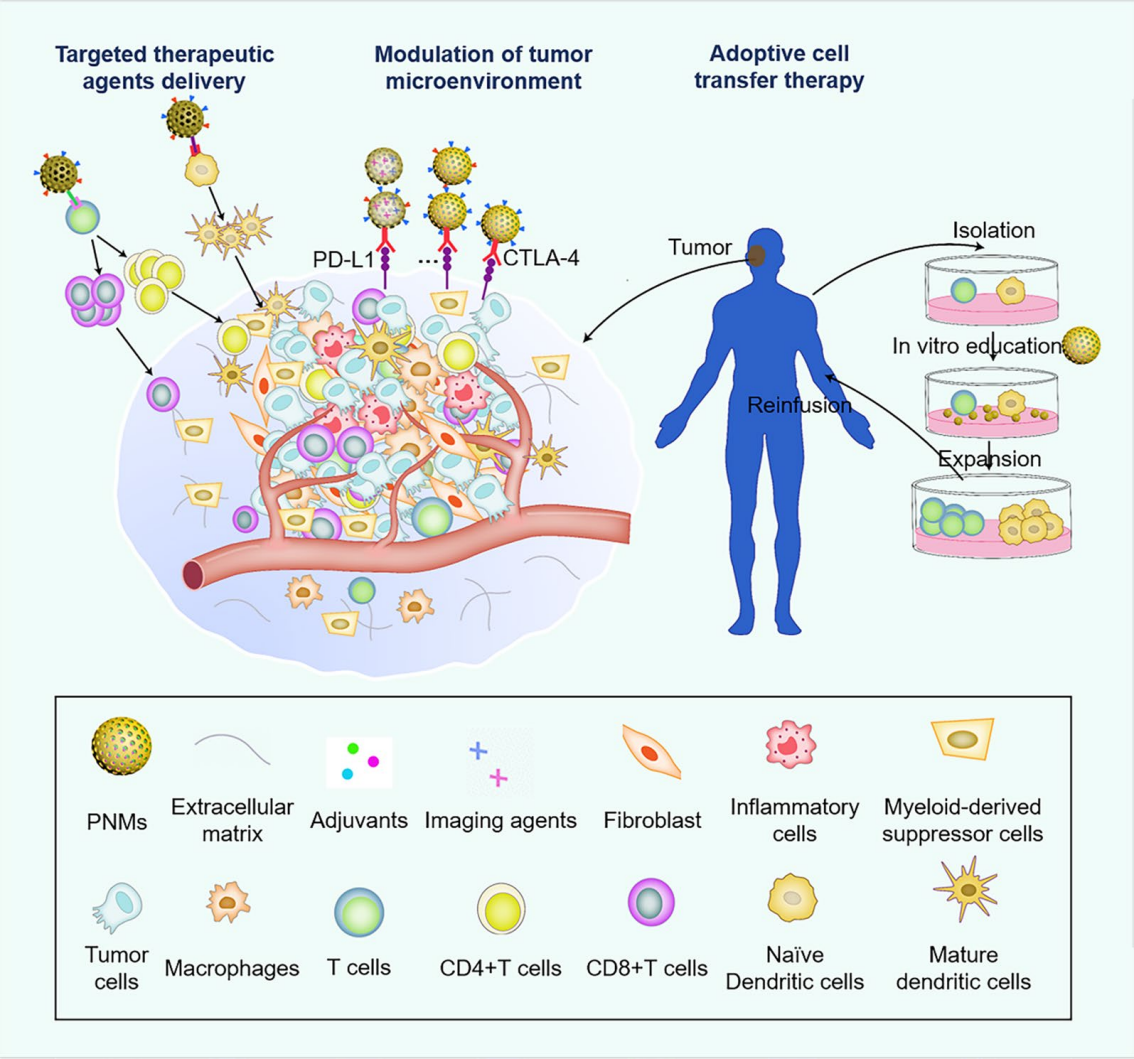
Schematic representation of three different strategies of porous nanomaterials used in cancer immunotherapy. Delivery of targeted therapeutic agents; modulation of the tumor microenvironment; and adoptive cell transfer therapy

Nanomaterials-based strategies provide new options and tools for cancer immunotherapy because of their unique biological and chemical properties [16]. Porous nanomaterials (PNMs) with porous structures and high surface/pore volume have been widely used in the biomedical field [17, 18], especially as drug carriers. PNMs possess some intrinsic advantages such as high loading capacity of biomolecules, tunable structures, abundant surface modification, and controllable release behavior of loaded molecules such as immunomodulators [11, 19, 20]. PNMs can enhance cancer immunotherapy through several pathways including delivering antigens and stimulating molecules into target cells/tissues, modulating immune dysfunction in the tumor microenvironment, and promoting ACT therapy efficacy (Fig. 2) [5, 21]. Additionally, PNMs can be engineered to combine cancer immunotherapy with other treatments such as PDT, PTT, or by acting as radiosensitizers [22, 23], to achieve better anti-cancer effects [24].

**Fig. 3:**
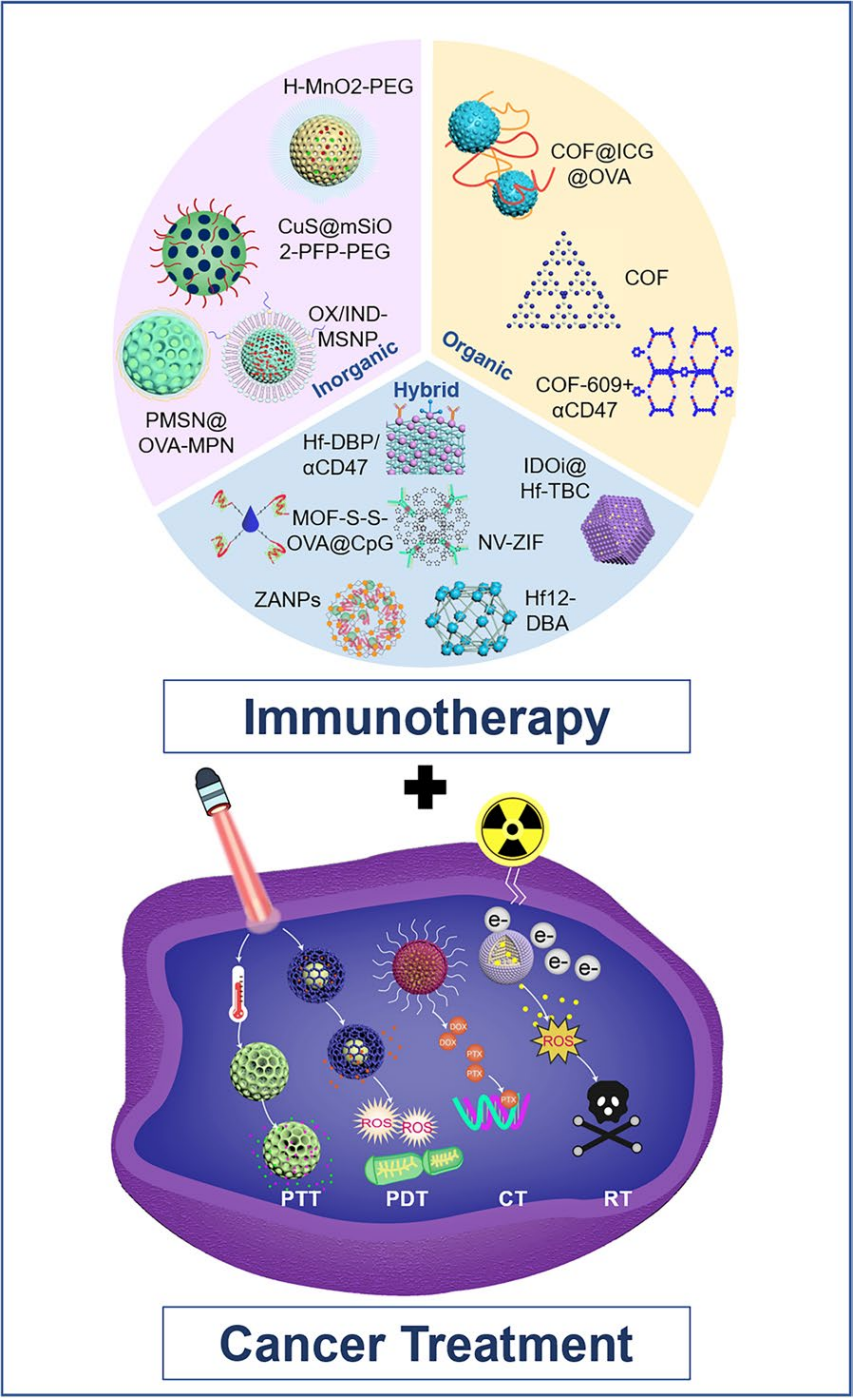
Schematic illustration of various inorganic, organic, and hybrid porous nanomaterials (PNMs) for tumor therapy. Inorganic PNMs include: H-MnO_2_-PEG [73], OX/IND-MSNP [55], PMSN@OVA-MPN [58], CuS@mSiO_2_-PFP-PEG [75]; organic PNMs include: COF@ICG@OVA 109], COF-609 + αCD47 [80]; hybrid PNMs include: Hf-DBP/αCD47 [98], IDOi@Hf-TBC [4], MOF-S-S-OVA@CpG [100], NV-ZIF [94], ZANPs [99], Hf12-DBA [96]. PNMs can achieve the combination of cancer immunotherapy with PDT, PTT, CT, and RT for better cancer treatment outcomes

In this review, we summarize the recent progress of employing PNMs for cancer immunotherapy, including delivery of targeted therapeutic agents, modulation of the tumor microenvironment and their application for adoptive cell transfer therapy. Based on chemical compositions, we divide PNMs into three categories: organic, inorganic, and hybrid PNMs (Fig. 3). For each type of PNM, the application for cancer immunotherapy and their performance are discussed, with a focus on how those PNMs are designed to address current barriers in conventional immunotherapy. Finally, the challenges and future directions of applying PNMs for cancer immunotherapy with potential better clinical outcomes are presented. It is expected that this review will provide useful guidance for the design of PNMs for efficient cancer immunotherapy with better performance.

## Advantage of PNMs for cancer immunotherapy

Nanoparticles have attracted much interest in cancer therapy due to the following advantages: nanomaterials-based DDSs with advantageous pharmacokinetics and pharmacodynamics, reduced drug toxicity [21]; excellent biocompatibility, low immunogenicity, high chemical, thermal and mechanical robustness [25]; the enhanced permeation and retention (EPR) effect [26], etc. PNMs are of special interest for cancer therapy due to their porous structure, they have the following features:

1. Porous nature: With pores, PNMs are widely used as drug reserves for various drugs for cancer immunotherapy. Based on the pore size, PNMs can be divided into three categories, i.e., microporous (pore size < 2 nm), mesoporous (2 nm < pore size < 50 nm), and macroporous (pore size > 50 nm) nanosystems [27]. Porosity endows nanomaterials with significant advantages. (i) A larger pore volume allows loading of multiple therapeutic agents with various purposes, triggering a series of therapeutic events [25, 28, 29]; (ii) Easily tuned aperture displays unique applicability. PNMs can be designed with a wide range of pore sizes (from 2 to dozens of nanometers), which enables the loading of different types of agents, from small drugs (chemotherapeutic agents) to larger molecules (proteins or oligonucleotide strands) [25].
2. PNMs can be engineered to present exquisitely controllable drug-release properties via placing stimuli-responsive pore blockers or sensitive hybrid coats on the surface of PNMs [25, 29].
3. Additionally, PNMs, such as porous silicon nanoparticles, have exhibited particular luminous characteristics [30–32], which can help us trace the whole process from drug loading to release and pharmacokinetic [33].

The last decades have witnessed encouraging progress of PNMs in drug delivery, bioimaging, biosensing, tissue engineering, and immunotherapy [34–39]. Recently, new PNMs such as covalent organic framework (COF) and metal–organic framework (MOF) are reported for the application of cancer immunotherapy [4, 40, 41]. Herein, we divide all PNMs into three catalogues based on the materials and present their application in cancer immunotherapy. They are inorganic, organic, and hybrid PNMs nanomaterials. Their properties and progress in cancer therapy are summarized in Table 1 and discussed in detail below.

**Table 1:** Summary of recent advances in porous nanomaterials for cancer immunotherapy

Strategies for immunotherapy barriers	PNMs	Composition	Target cells	Main results	Ref.
Inorganic PNMs					
Reversing immunosuppressive tumor microenvironment	MSNPs	OX/IND-MSNP	Tumor cells and APCs	A nano-enabled approach for OX and IND delivery to the PDAC site can be used for an immunotherapy response premised on the induction of ICD plus reversal of IDO immune suppressive effects	[55]
	Fe_3_O_4_ nanoparticles	Fe_3_O_4_-OVA nanocomposites	BMDC and macrophages	A nanopotentiator stimulated the maturation of BMDCs and the activation of T cells and macrophages for the subsequent inhibition of the growth and metastasis of tumors	[67]
	DOX NPs, (shPD-L1 + Spam1) NPs	DOX NPs and (shPD-L1 + Spam1) dual-gene codelivery NPs	Tumor cells and DCs	Immune cocktail therapy was constructed, and the nanocomposites achieved multiple activations of the cancer-immunity cycle by synergistic effects of ICT and chemotherapy	[106]
Tumor-targeted delivery	PSiNP	PSiPs-HER-2	Tumor cells	PSiPs-HER-2 achieved specific targeting and destruction of breast cancer cells in vitro	[63]
	PHNPs	PHNPs@DPA-S-S-BSA-MA@3-MA	TAMs	PHNPs@DPA-S-S-BSA-MA@3-MA showed good efficiency for targeting TAMs, activating immune responses, and inhibiting tumor growth in vivo	[51]
	MSNs	Carbon nanodots-based MSNs (CD@MSNs)	NK cells, macrophages	Biodegradable CD@MSNs combined with PTT could specifically accumulate in the tumor sites and effectively inhibited tumor metastasis	[56]
	MSN	MSN@polyphenol	Tumor cells	Highly biocompatible and biodegradable polyphenol-coated MSNs can achieve controlled molecule release	[57]
Enhancing uptake and presentation	PSi	LPSiNPs	B cells	Engineered nanoparticles working with the immune system enhanced the activation of APCs and B cells	[34]
	PMSN	PMSN@OVA-MPN	DC2.4 cells	PMSN@OVA-MPN promoted the OVA uptake by DC2.4 cells and enhanced tumor-specific cellular immune response for effective inhibition of tumor growth	[58]
	IMHCSs	IMHCS-OVA	APCs	OVA-loaded IMHCSs enhanced uptake in APCs and induced the maturation of APCs	[59]
Achieving multi-functionality	Mesoporous MnO_2_ nanoshells	H-MnO_2_-PEG/C&D	Tumor cells	Novel H-MnO_2_-PEG/C&D as a multifunctional theranostic platform modulated TME and chemo-PDT therapy further enhanced immunotherapy	[73]
	MSRs	MSRs loaded with GM-CSF, CpG, and OVA	BMDC	Injectable MSRs provided a 3D microenvironment and may serve as a multifunctional vaccine platform to modulate host immune cell function and provoke adaptive immune responses	[60]
	CuS bMSN	CuS@mSiO_2_-PFP-PEG (CPPs)	Tumor cells	Multifunctional nanoplatform CPPs achieved photoacoustic and ultrasound dual modality-guided PTT combined immunotherapy	[75]
	bMSN	bMSN (CpG/Ce6)	DCs	Biodegradable MSN vaccination is a promising platform for personalized cancer immunotherapy via the combination of imaging and PDT	[61]
	PDA NPs	PDA-MB@MnO_2_	Tumor cells	A safe and effective nanosystem for metastatic breast cancer treatment by the combination of supplemental oxygenation with multi-modal imaging-guided phototherapies	[107]
	Pristine PLGA NPs	CNP	Tumor cells	Uniform-sized CNP significantly elevated the internalization efficiency of exogenous GM-CSF and IL-2 by tumor cells	[108]
	FeSe_2_ nanoflower	FeSe_2_-PE	Tumor cells	The FeSe_2_-PEG nanoflowers were fabricated to achieve the on-demand release of H_2_Se on NIR-II photoactivation to fight against breast cancer	[62]
Organic PNMs					
Achieving multi-functionality	COF	COF@ICG@OVA	DCs	Combined with NIR irradiation and a checkpoint inhibitor, multi-functional COF@ICG@OVA suppressed tumor growth and metastasis by ROS and hyperthermia	[109]
	COF	COF-609 + αCD47	Tumor cell	The study offered the first integration of PDT and immunotherapy by 3D COFs to inhibit cancer metastasis and recurrence and demonstrated a new way to design ICD inducers	[80]
Hybrid PNMs					
Reversing immunosuppressive tumor microenvironment	MOF (MIL-100)	MIL-100 with MTO, hyaluronic acid	CT26 cells	Robust antitumor immunotherapy by combining PTT with chemotherapy to enhance ICD and inhibited the activity of the immunosuppressive cells in TME	[92]
	MOF	MOF-OVA@CpG	APCs	Co-delivery of antigen and CpG showed significant T cell activation and cytokine release, and successful suppression of tumor growth	[93]
	Biomimetic MOFs	NV-ZIF nivolumab	PBMCs	NV-ZIF showed a higher efficacy to activate T cells in hematological malignancies. Modified by coating with CCM to enable tumor-specific targeted delivery	[94]
	ZIF-8 NPs	ZIF-8/CpG ODNs	RAW264.7 cells	ZIF-8/CpG ODNs showed no cytotoxicity and promoted the uptake of CpG ODNs in RAW264.7 cells, which further increased the secretion of immune cytokines	[95]
	Hf-based nMOFs	Hf12-DBA	CT26 cells	The combination of nMOF-mediated RT and PD-L1 ICB achieved effective T cell proliferation, enhanced tumor infiltration, and inhibition of the distant tumors	[96]
	Hybrid Nanocarrier	Ce6/MLT@SAB	Tumor cells	Ce6/MLT@SAB-mediated PDT combined with ICB therapy further upregulated the numbers of CD4^+^ and CD8^+^ T cells in tumor sites and decreased the level of MDSCs	[97]
	nMOFs	IMD@Hf-DBP/αCD47	Macrophages, tumor cells	Under X-ray irradiation, IMD@Hf-DBP/αCD47 modulated the immunosuppressive TME and activated immune events when synergized with an ICB therapy	[98]
Tumor-targeted delivery	MOFs	CpG/ZANPs	APCs	The first facile, green synthesis of aluminium-integrated CpG/ZANPs targeted lymph nodes, and their cargo was internalized by APCs, significantly suppressing tumor growth	[99]
	Calcium phosphate NPs	LCP-II NPs	Tumor cells	The novel NP composites effectively delivered siRNA to tumor sites in a xenograft model and improved the tissue distribution and uptake by tumor tissues	[110]
Enhancing uptake and presentation	MIL-101-Fe-NH2 NPs	MOF-S-S-OVA@CpG	APCs	MOFs can improve the uptake of OVA by APCs and show promising application in the codelivery of antigens and immune adjuvants	[100]
	Cationic nMOF	W-TBP/CpG/α PD-L1	DCs	Cationic W-TBP combines PDT and CpG delivery to enhance antigen presentation	[101]
	Zirconium-based MOF	UiO-OVA	APCs	UiO-OVA can produce forceful antigen-mediated humoral immunity and effectively activate T lymphocyte proliferation	[102]
Achieving multi-functionality	MOF	MOF-OVA@CpG	APCs	Co-delivery of antigen and CpG showed significant T cell activation and cytokine release, and successful suppression of tumor growth	[93]
	nMOFs	IMD@Hf-DBP/αCD47	Macrophages, tumor cells	NMOFs can co-deliver multiple immunoadjuvants for macrophage therapy to boost systematic immune responses an antitumor efficacy by the combination of RT-RDT	[98]
	Cuporphyrin nMOF	Cu-TBP	B16F10 cells	Cu-TBP-mediated CDT/PDT elicited systemic antitumor immune responses via triggering innate immune responses and re-activating T cells in primary and metastatic tumors	[103]
	nMOF	TBP-nMOF	4T1 cells	PDT mediated by TBP-nMOF in combination with αPD-1 ICB therapy can suppress the growth of the primary tumor and metastatic tumor	[104]
	MOFs	TPZ/UCSs	CT26 cells	TPZ/UCSs improved cancer treatment efficiency via the combination of NIR light-induced PDT and hypoxia-activated chemotherapy, which enhanced tissue penetration in PDT	[105]

## Inorganic porous nanomaterials for cancer immunotherapy

Inorganic PNMs are considered “value-added” materials owing to their unique size-related and quantum-constrained features that, to some extent, account for the great interest in nanomaterials-based immune-related applications [42]. Inorganic PNMs possess large and tunable surface areas, surface functionalization [43], biocompatibility, thermal and mechanical robustness, as well as an exquisitely controlled drug release behaviour, which renders them qualified candidates for biomedical applications [25]. Additionally, inorganic NPs show a bright prospect for many fields, such as imaging [44], catalysis [45], sensing [46], and drug delivery [47].

Common inorganic materials are Au nanoparticles [48], porous silicon nanoparticles [49], mesoporous silica nanomaterials (MSNs) [50], carbon nanoparticles, ion oxide nanoparticles [51], Au@Rh core–shell nanoparticles [52], CeO_2_ nanoparticle [53], Pt spiral [54] etc. The inorganic PNMs are usually prepared by sol–gel method (such as MSNs [55–61]), hydrothermal method (such as iron oxide nanoparticles [51, 62]), chemical vapour deposition method [63] and electrochemical etching method (such as porous silicon nanoparticles [34]).

### Reversing the immunosuppressive tumor microenvironment

The immunosuppressive tumor microenvironment includes suppressive components, insufficient immune cells infiltration, and soluble factors [15, 64], which supports tumor progression and metastasis and restricts the function of infiltrating APCs and T cells, and poses great challenges for cancer treatment [65]. The strategies of cancer immunotherapy based on nanomaterials include altering the immunoreactivity within the primary tumor, boosting the immune system, compromising the pre-metastatic niches and ultimately inhibiting the formation of secondary metastatic lesions [66].

Inorganic PNMs-based strategy can reverse the immunosuppressive tumor microenvironment by simply recruiting more anti-tumor immune cells, such as cytotoxic T cells. For example, MSNs were used to load and deliver immunogenic cell death (ICD)-inducing chemotherapeutic agent, oxaliplatin and IDO inhibitor, indoximod. The oxaliplatin and indoximod loaded MSNs effectively induced innate/adaptive anti-pancreatic ductal adenocarcinoma immune responses and resultant tumor repression, which was accomplished by the recruitment of cytotoxic T cells and simultaneous suppression of Foxp3^+^ T cells [55]. Iron oxide nanoparticles were also used to deliver ovalbumin (OVA) to stimulate the maturation of bone marrow-derived dendritic cells and the activation of T cells and macrophages. Consequently, the growth and metastasis of tumors were effectively inhibited [67].

### Tumor-targeted delivery

Targeted therapies can be achieved by direct and indirect approaches: the former involves changing cell-specific signal events (by antibodies or small molecules inhibitors) [68], the latter refers to using molecular targets, overexpressed or exclusively expressed on the surface of tumor cells, to send cytotoxic molecules (such as chemotherapeutic drugs and toxins). With targeting, higher concentrations of therapeutic agents in tumor sites can be achieved to reduce the toxicity and side effects [69, 70]. For example, Fenollosa et al. synthesized porous silicon particles conjugated with a specific antibody (HER-2) for breast cancer treatment, which showed specific targeting and destruction of tumor cells in vitro and in vivo [63]. In a recent study, porous hollow iron oxide nanoparticles (PHNPs) were synthesized to load 3-MA (a P13K γ small molecule inhibitor) and further modified by mannose for TAMs targeting. The functional nanoparticles demonstrated high efficacy in targeting TAMs, resulting in enhanced anti-tumor immunotherapy by an intracellular switch of the TAM phenotype [51]. Qian et al. synthesized biodegradable MSNs by incorporating polymer-coated carbon nanodots into the ordered framework of mesoporous silica nanoparticles (CD@MSNs). CD@MSNs can not only increase the photothermal effect and targeted gathering but suppress the metastasis by enhancing the anti-tumor immune responses [56]. Polyphenol-coated porous nanomaterials (MSN@polyphenol) were developed by in situ self-polymerization method. MSN@polyphenol improved stability, reduced drug leakage, and can be easily functionalized for targeting [57].

### Enhancing antigens uptake and presentation

Poor immunogenicity usually leads to non-responsive or low-responsive tumor immunotherapy [15]. Shifting non-responsive tumors into responsive tumors by enhancing the tumor immunogenicity can enhance the therapeutic effects. Improving the efficiency of antigen uptake and presentation is a common approach [71]. Several strategies can be carried out to enhance antigens uptake and presentation, such as elevating the concentration of antigens, activating APCs and promoting antigens uptake in APCs. Luminescent porous silicon nanoparticles contained copies of an agonistic antibody (FGK45) to the APC receptor, which significantly promoted the activation of B cells and APCs, and triggered stronger immune responses than free FGK45 [34]. In another work, pH and reduction dual responsive MSNs were designed to deliver OVA (PMSN@OVA-MPN) and release them inside tumor cells. PMSN@OVA-MPN elevated OVA internalization by DC2.4 cells as well as the release of antigens from the lysosome, eliciting stronger cellular immune responses for more effective inhibition of tumor progression [58]. Mono-dispersed mesostructured hollow carbon spheres are also used for OVA delivery and demonstrated good drug-loading efficacy, sustained-release behavior, enhanced cellular uptake and promoted APCs maturation [59].

### Achieving multifunctionality

Poor immunotherapy may result from multiple aspects, such as low antigen concentration, insufficient antigen identification, and poorly controllable drug release mechanisms. Multifunctional inorganic PNMs may simultaneously deliver different types of therapeutic agents (such as neoantigens, adjuvant, photosensitizer and imaging agents). This combination of immunotherapy with other approaches, such as chemotherapy, PTT and PDT can achieve synergic effects. For example, imaging-guided PTT with therapeutic agents can provide a more efficient option for tumor metastasis inhibition, especially for metastatic lymph nodes and large solid tumors [72].

**Fig. 4:**
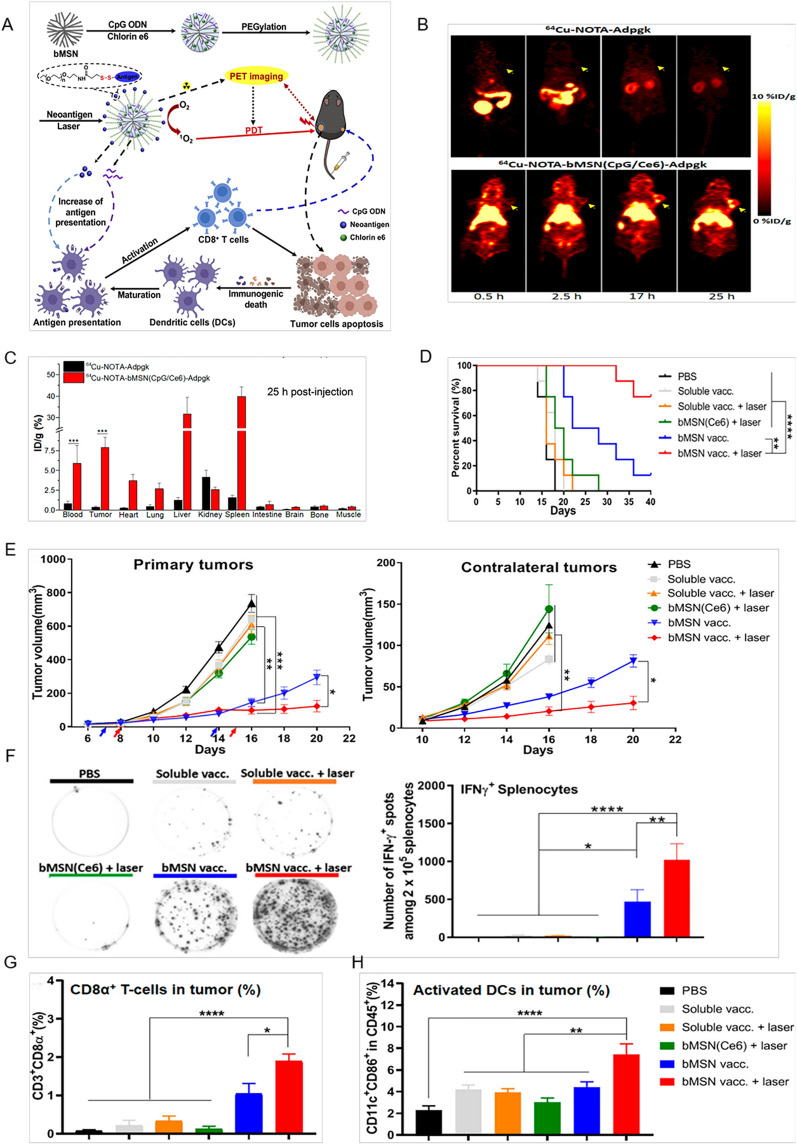
Inorganic porous nanomaterials for tumor immunotherapy. **A** Schematic illustration of synthesis of bMSN (CpG/Ce6)-neoantigen and mechanism of the composite as nanovaccines for PDT-mediated immunotherapy. **B** Serial PET images of MC-38 tumor-bearing mice at different time points postinjection of 64Cu-NOTA-Adpgk or 64Cu-NOTA-bMSN (CpG/Ce6)-Adpgk. Tumors are indicated by yellow arrowheads. **C** Biodistribution of 64Cu-NOTA-Adpgk and 64Cu-NOTA-bMSN (CpG/Ce6)-Adpgk in MC-38 tumor-bearing mice at 25 h postinjection. **D** Overall survival curves of each group. **E** Average primary and contralateral tumor growth curves of each group. **F** On day 21, IFN-γ ELISPOT assay was conducted by ex vivo restimulation of splenocytes with M27 and M30 peptides at a concentration of 10 μg/ml. Meanwhile, tumor tissues were analyzed for the frequencies of CD3^+^CD8α^+^ T-cells (**G**) and CD11c^+^CD86^+^ DCs (**H**) by flow cytometry (Adapted with permission from [61]. Copyright © 2019 American Chemical Society)

MnO_2_ nanomaterials modified with polyethylene glycol can achieve the co-loading of chlorine e6 (a photodynamic-related molecule) and DOX. The nanocomposite H-MnO_2_-PEG/C&D released therapeutic agents under lower pH and induced H_2_O_2_ degradation to alleviate the hypoxic tumor microenvironment. Remarkable anti-tumor immune effects were achieved through combining chemo-photodynamic therapy with ICB therapy [73]. Mesoporous silica rods (MSRs) also worked as a 3D biomimetic microenvironment for immune cells. MSR-based vaccine elevated the serum antibody level of T helper type 1 cells, type 2 cells, and cytotoxic T cells for more effective immunotherapy [60, 74]. In another work, a multifunctional platform was established based on biodegradable MSNs and neoantigens, cytosine-phosphate-guanine oligodeoxynucleotides adjuvant and photosensitizer chlorin e6 were simultaneously combined and loaded. This nanosystem showed a specific accumulation in tumor sites and can be applied for cancer management in combination with positron emission tomography (PET)-guided PDT [61] (Fig. 4).

In addition to one composite nanomaterial, complex nanoparticles such as core–shell contracture are also fabricated for cancer immunotherapy. For example, core–shell CuS@mSiO_2_-PFP-PEG nanoparticles were synthesized with good biocompatibility, photoacoustic/ultrasound imaging and a strong PTT effect. The multifunctional core–shell CPP can not only eradicate primary lesions but also suppressed the formation of secondary metastases by the combination of PTT and PD-1 ICB therapy [75].

## Organic porous nanomaterials for cancer immunotherapy

**Fig. 5:**
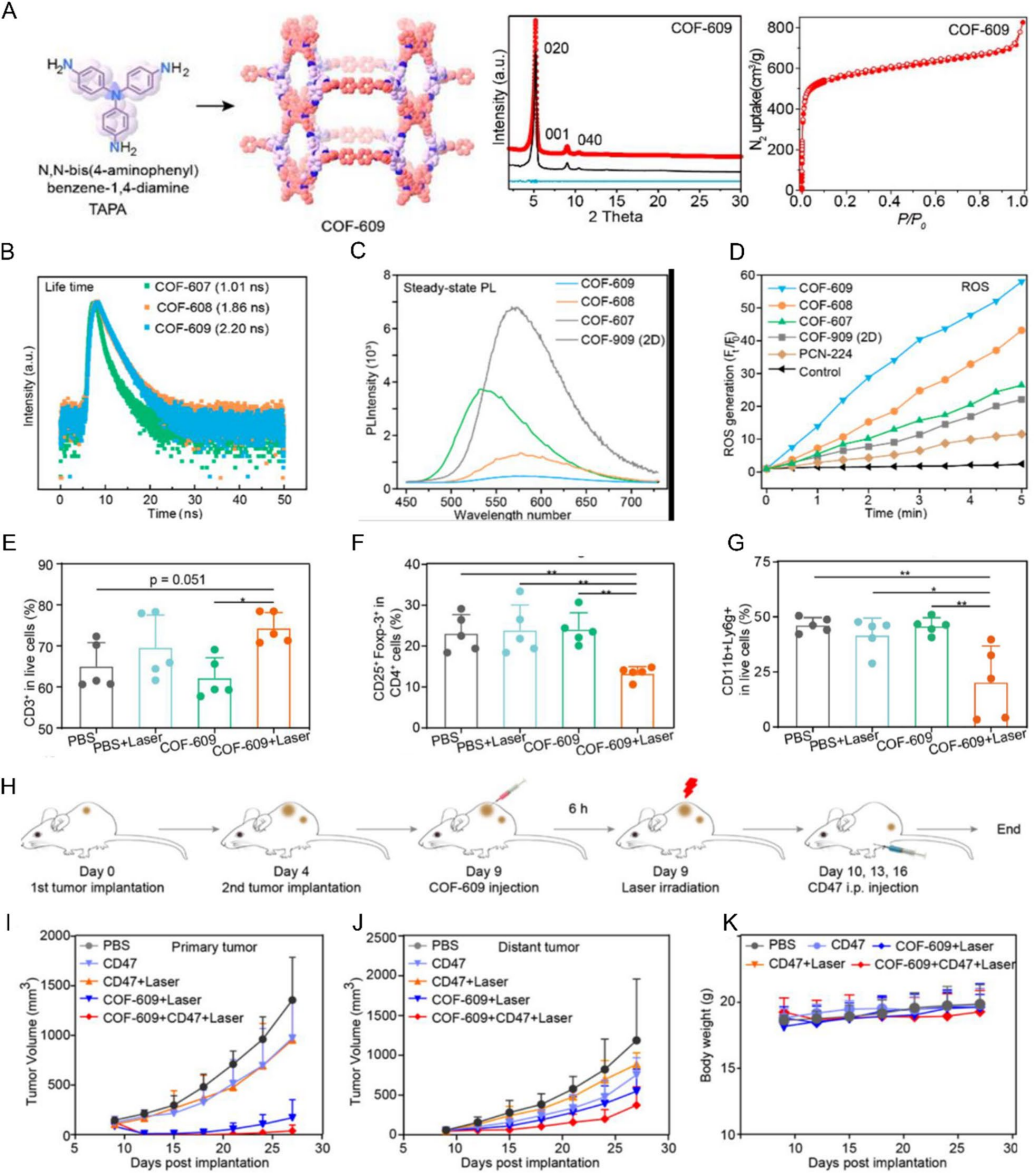
Organic porous nanomaterials for tumor immunotherapy. **A** Construction and characterization of three-dimensional (3D) covalent organic frameworks (COFs). **B** Time-resolved PL spectra of COF-607 to COF-609. **C** Steady-state PL spectra contrast of COF-607 to COF-609. **D** ROS production efficiency of COF-607, COF-608, and COF-609 compared to PCN-224. **E** Quantification of immune cells in the draining lymph nodes, CD3^+^ T cells (**E**) and CD4^+^ CD25^+^ Foxp3^+^ Treg cells (**F**). **G** Quantification of CD11b^+^ Ly6g^+^ MDSCs in the spleen. **H** Time schedule of the establishment of bilateral tumor mouse model and treatments. **I**, **J** Growth curves of primary and distant tumors of bilateral 4T1 tumor-bearing mice. **K** Body weight of 4T1 tumor-bearing mice with different treatments (Adapted with permission from [80]. Copyright © 2021 The Authors. Published by American Chemical Society)

Though organic nanomaterials such as lipid nanoparticles and polymer nanoparticles are widely used for cancer immunotherapy, porous organic nanomaterials are rarely reported until recently due to the difficulty of fabrication. A type of organic framework with porous structure, COFs, has been reported recently with tunable pore size and large surface area [76]. COFs draw special attention for their favourable biocompatibility, porosity, structural uniformity, comprehensive functionality, and synthesis flexibility [27]. Diversified organic PNMs, such as COF LZU-1, CMP, APTES-COF-1, and CTP, have been constructed for various biomedical applications [77]. COFs have been employed for some cancer treatments such as imaging, photoacoustic tomography, PTT, PDT, etc. [41, 78, 79]. COFs are also used for cancer immunotherapy recently. For example, COF@ICG@OVA NPs were fabricated by embedding indocyanine green (ICG) inside and coating OVA on the surface. The COF@ICG@OVA NPs triggered systemic immune responses and suppressed neoplasm metastasis by combining PD-L1 ICB therapy with PTT and PDT. A recent study reported that the optical properties of modified COFs could be adjusted to yield excellent reactive oxygen species generation via linking ICD inert monomers into the COF backbone. Another type of COF can function as an ICD inducer to elicit powerful and long-lasting immune responses [80] (Fig. 5). The COF was synthesized by simple self-assembling methods by adding triple-topic amine building blocks into a tetra-topic aldehyde, tetrabenzaldehyde [80]. The application of COFs for cancer immunotherapy remains in its early stages.

## Hybrid porous nanomaterials for cancer immunotherapy

MOFs are generally built of a class of solid porous materials, which comprise inorganic metal ions or metallic clusters acting as nodes, and organic ligands as bridges between the nodes [81–83]. Highly structural variability, improved biocompatibility, ease of surface functionalization, as well as large surface area make MOFs attractive for cancer immunotherapy [82, 84]. MOFs are usually synthesized through the self-assembling of metal–oxygen clusters and organic linkers followed by crystallization. MOFs usually have the following advantages: (1) greatly tunable properties and higher drug loading capacity, (2) controllable multifunctionality [85], (3) flexible metal–ligand bonds make sure that MOFs can be degraded at expected sites, which showed greatly controllable therapeutic agents release [86]. MOFs have been extensively used as heterogeneous catalysts for bacteria inhibitors [87], wound healers [88], diagnostic agents [89], radiosensitizers [90], and applied in PET imaging as well as targeted chemotherapy [91]. For cancer immunotherapy, MOFs mainly work as nanocarriers to deliver therapeutic agents to target sites.

### Reversing the immunosuppressive tumor microenvironment

As mentioned above, the immunosuppressive tumor microenvironment leads to poor immunotherapy efficiency, with immunosuppressive cells (such as MDSCs, Treg cells, and M2 macrophages) being the major culprits. Given the large accumulation of immunosuppressive cells and the insufficient infiltration of immunoreactive cells, targeted strategies based on inorganic–organic PNMs to reverse the immunosuppressive tumor microenvironment are needed. Ni and co-workers prepared MOF (MIL-100) to load chemotherapy agents mitoxantrone and hyaluronic acid and conjugated it with a targeting molecule (anti-OX40 antibody) on the surface. These multifunctional nanoparticles reversed the immunosuppressive tumor microenvironment by suppressing the function of immunosuppressive cells, such as M2 macrophages, MDSCs and regulatory T cells and achieving very high antitumor efficacy [92]. Duan et al. fabricated a dual-delivery of antigens and immunostimulatory molecules platform based on MOFs. This nanocarrier demonstrated enhanced antitumor effects in B16-OVA melanoma via the recruitment of tumor-killing immunocytes [93]. In another work, zeolitic imidazolate frameworks (ZIFs), one type of MOF, were designed to target deliver Nivolumab, a monoclonal antibody ICB drug approved by FDA in a controlled release manner. ZIFs were synthesized by mixing zinc nitrate with 2-methylimidazole at room temperature, followed by ultrasonication. Nivolumab loaded ZIF elevated the efficacy to activate T cells and achieved better antitumor performance [94]. ZIF-8 was also used to load and deliver CpG ODNs with improved internalization efficacy by immune cells, resulting in stronger stimulated immune responses for immunotherapy [95]. Hf-based nMOFs were applied as radioenhancers for more effective and safer RT, and the α-PD-L1 antibody reversed the immunosuppressive tumor microenvironment.

**Fig. 6:**
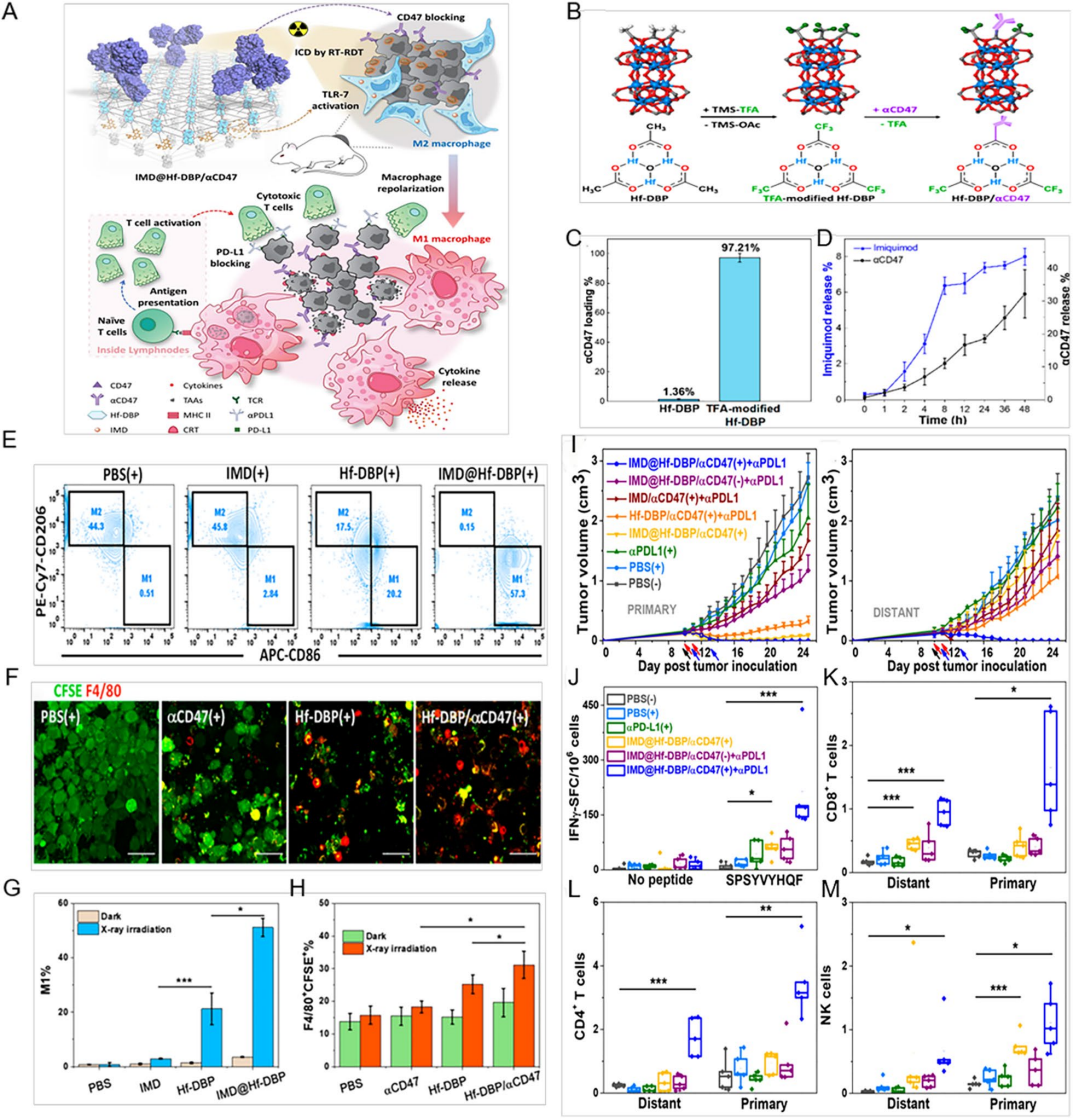
Inorganic–organic hybrid porous nanomaterials for tumor immunotherapy. **A** Illustration showing repolarization of M2 to M1 macrophages and promotion of phagocytosis via blocking the “don’t-eat-me” signal on the surface of tumor cells by IMD@Hf-DBP/αCD47 with X-ray radiation. **B** Surface modification of Hf-DBP for αCD47 loading. **C** αCD47 loading efficiency of Hf-DBP and TFA-modified Hf-DBP. **D** Release profiles of IMD and αCD47 of IMD@Hf-DBP/αCD47, n = 3. **E** Repolarization of macrophages cocultured with CT26 cells treated with PBS (+), IMD (+), Hf-DBP (+), or IMD@HfDBP (+). **F** Phagocytosis of CFSE-labeled CT26 cells treated with PBS (+), αCD47 (+), Hf-DBP (+), or Hf-DBP/αCD47 (+) by macrophages observed under CLSM, scale bar = 50 μm. Quantification of macrophage repolarization (**G**) and phagocytosis (**H**), n = 3. *P < 0.05, **P < 0.01, and ***P < 0.005 from control. **I** Growth curves of primary tumors and distant tumors of bilateral CT26 tumor-bearing mice. Black, red, and blue arrows represent intratumoral injection, X-ray irradiation, and intraperitoneal injection, respectively. **J** ELISpot assay to measure IFN-γ generating T cells with tumor-specific responses in splenocytes after treatments. The percentage of tumor-infiltrating CD8^+^ cells (**K**), CD4^+^ T cells (**L**), and NK cells (**M**) in the total number of tumor cells. n = 5. *P < 0.05, **P < 0.01, and ***P < 0.005 from control (Adapted with permission from [98]. Copyright © 2020 American Chemical Society)

Furthermore, the combination of RT and PD-L1 ICB therapy achieved higher efficiency of RT with minimal side effects and initiate immunotherapy for non-immunogenic tumors [96]. Ce6/MLT@SAB-mediated PDT combined with ICB therapy further enhanced antitumor outcomes by upregulating the quantity of CD4^+^ and CD8^+^ T cells and decreasing the level of MDSCs in tumor sites [97]. Ni et al. developed IMD@Hf-DBP/αCD47 by loading a toll-like receptor 7 agonists, IMD, and anti-CD47 antibody to one type of MOF (Hf-DBP nMOF). IMD@Hf-DBP/αCD47 boosted immune treatment responses in that anti-CD47 antibody reversed immunosuppressive tumor microenvironment and IMD converted immunosuppressive M2 macrophages to immunostimulatory M1 macrophages [98] (Fig. 6).

### Tumor-targeted delivery

MOFs are also surface engineered with active targeting molecules to achieve better efficiency and lower toxicity. Nivolumab loaded ZIF-8 was coated with cancer cell membranes and demonstrated to have improved tumor-specific recognition and achieved tumor-targeted delivery of agents [94]. MOFs are also modified with a Toll-like receptor 9 agonist CpG and the nanomaterials presented specific targeting of lymph nodes. The surface-modified MOFs triggered enhanced antigen-specific immune responses that greatly suppressed tumor growth with minimal cytotoxicity [99].

### Enhancing antigens uptake and presentation

Designing nanocarriers with improved antigen uptake and presentation ability can enhance the efficacy of immunotherapy. Yong et al. developed a biodegradable MOF (MIL-101-Fe-NH_2_) and co-delivered OVA and CpG. They demonstrated that MIL-101-Fe-NH_2_ with appropriate size improved the antigen immunogenicity thus enhancing antitumor immune responses [100]. Ni and co-workers prepared a new cationic MOF (W-TBP) to deliver CpG oligodeoxynucleotides to DCs with high efficacy. In addition, W-TBP also enabled PDT and the synergistic effects resulted in expansion and activation of cytotoxic T cells, resulting in > 97% tumor regression in a bilateral breast cancer model [101]. In another study, amino-functionalized zirconium-based MOFs (UiO-AM) were used as nanocarriers for the efficient uptake of antigen OVA by APCs (UiO-OVA) and promoted the maturation of APCs to enhance innate and adaptive immunity [102].

### Achieving multifunctionality

As a hybrid material, MOF possesses the properties of both organic and inorganic PNMs. Multifunctionality can be achieved by MOFs through the combination of cancer immunotherapy with other treatment methods, such as PDT, PTT and chemotherapy. A Cu-porphyrin nMOF utilized Cu^2+^ to catalyze E2-driven chemodynamic therapy and light-triggered PDT to achieve local tumor therapy in a mouse model with high E2 expression tumors. In addition, this Cu-porphyrin nMOF also provided the possibility of eliciting systemic antitumor immune responses in hormonally dysregulated tumors with the combination of ICB therapy [103]. Benzoporphyrin-based MOF (TBP-MOF) was also applied as a PDT-enhancer with high chemical stability and improved photophysical property to suppress the growth of tumors [104]. Core-shelled nanoparticle@porphyrinic MOF was also developed and hypoxia-activated prodrug tirapazamine (TPZ) was encapsulated inside the pores. This multifunctional MOF exhibited stronger cancer treatment efficacy by combining NIR radiation-enhanced PDT with hypoxia-boosted chemotherapy [105].

## Conclusion and outlook

Nanomaterials could address the challenges in traditional DDSs and offer novel options to trigger stronger immune responses for cancer immunotherapy. Compared with conventional DDSs, PNMs possess unique properties for antitumor immunotherapy, such as high loading capacity of immune-related biomolecules and co-delivery of multiple therapeutic agents, good biocompatibility, high stability, low immunogenicity and cells or tissue targeting. Based on the nature of the materials, the PNMs can be divided into three catalogues and the typical examples, advantages and disadvantages of those 3 PNMs are summarized in Table 2.

**Table 2:** Advantages and disadvantages of three types of PNMs

Types	Inorganic nanomaterials	Organic nanomaterials	Hybrid nanomaterials	Ref.
Typical example	MSNs, mesoporous silicon NPs, mesoporous carbon	COFs	MOFs	[27]
Advantages	Good biocompatibility; ease of functionalization, high drug loading capacity, and some unique physicochemical properties such as optical, magnetic, electrical, ultrasonic, and catalytic properties	Good biocompatibility; biodegradability; controllable particle size; different functionalization	Advantages of both organic and inorganic material; improved biocompatibility; biosensing, high catalytic activity, optical properties and so on	[84, 111–115]
Disadvantages	Poor biodegradability and accumulation of metal ions may have potential toxicity	Limited pore size; few reports about the degradability	Possible toxicity needs further investigation; limited pore size; few reports about the degradability	(116, 117)

Despite PNMs showing favorable properties for cancer immunotherapy, several challenges remain that need future exploration.

1. Standard operating procedure of the fabrication and characterization methods to test the stability and reproducibility of PNMs, which can potentially facilitate their translation [27]. Manufacturing methods that allow large-scale production of PNMs with minimum batch-to-batch discrepancy are required.
2. The biodegradability, toxicity and interaction of PNMs with the immune system still need more exploration. For inorganic PNMs, the degradation rates are usually too slow and not desired. Introducing organic molecules into the inorganic framework can be a useful strategy to change the degradation rate [63].
3. In vivo studies of the pharmacokinetics and efficiency of PNMs are needed to further evaluate their safety and biocompatibility in more comprehensive models that better simulate the pathophysiological states of human beings, especially in large animal models.
4. The target delivery of drugs/therapeutic agents is still difficult to achieve and represents a big obstacle that limits cancer treatment results [118]. Multifunctional PNMs with rational designs can control the release of the drug at specific sites in a more precise and efficacious manner and can be a promising solution. Many strategies can enhance tissue penetration for more effective immunotherapy of PNMs, such as remodelling of the tumor microenvironment [119], charge inversion [120], dimensional change, surface modifications [121], and tissue-penetrating surface modification [122].
5. PNMs based cancer immunotherapy can combine with other treatment strategies, such as PDT, PTT, chemotherapy, RT and immune cocktail therapy to further enhance the anti-tumor efficacy.
6. It is interesting to note that the recent nanorobots are developed for various medical applications such as diagnosis, imaging and intervention [123]. Those nanorobots usually can be driven in a controlled manner and the whole process inside the body can be monitored. The development of nanorobots inspired us to desire more smart PNMs in the future that are equipped with the ability of guided motion, real-time tracking (imaging) and deliver drugs in a controlled manner. With smaller sizes and much lower costs compared to nanorobots, PNMs have a broader application in cancer immunotherapy. Additionally, some smart artificial immune cells, such as artificial APCs (aAPCs) [124], have been applied for cancer immunotherapy. AAPCs have been exploited as a versatile platform for cellular therapies including antigen-specific CD8^+^ T cells, antigen-specific CD4^+^ T cells, CAR-T cells, Treg cells, NK cells, etc. AAPCs facilitate the delivery of essential signals to selected subsets of T cells [125] and rapidly expand tumor-specific T cells [126]. Inspired by artificial immune cells, PNMs with multifunctions similar to a cell or cell component are also promising with more specific & efficient functions and avoid the potential risks of allergic reactions.

In summary, PNMs have demonstrated the potential of overcoming the barriers of current cancer immunotherapy and enhancing the anti-cancer efficacy. With a unique porous structure, PNMs can load a large amount of immunotherapy related biomolecules, deliver them in a targeted manner, modulate the tumor microenvironment and regulate the immune cell function. Although a great stride has been made to facilitate the advances in PNMs for cancer immunotherapy, the applications of porous nanostructures in clinical practice remain in a fledging period. With interdisciplinary cooperation and cumulative knowledge reserve, inspiringly, the progress will be accelerated with expected breakthroughs in porous nanostructures for cancer immunotherapy. PNMs are promising and will play an increasingly important role in the field of cancer immunotherapy.

## Data Availability

All data generated or analyzed during this study are included in this published article and the Additional Information.

## Abbreviations

PNMs: Porous nanomaterials
DDSs: Drug delivery systems
ICB: Immune checkpoint blockade
PD-1: Programmed death protein 1
PD-L1: Programmed death-ligand 1
CTLA-4: Cytotoxic T-lymphocyte-associated protein 4
ACT: Adoptive-cell-transfer
PDT: Photodynamic therapy
PTT: Photothermal therapy
APC: Antigen-presentation cells
NPs: Nanoparticles
FDA: Food and Drug Administration
EPR effect: Enhanced permeation and retention effect
pSiNPs: Porous silicon nanoparticles
COF: Covalent organic framework
MOF: Metal–organic framework
PSiP: Porous silicon particles
MSNs: Mesoporous silica nanoparticles
MDSCs: Myeloid-derived suppressor cells
Tregs cells: Regulatory T cells
TAMs: Tumor-associated macrophages
ICD: Immunogenic cell death
PHNPs: Porous hollow iron oxide nanoparticles
OVA: Ovalbumin
CPPs: CuS@mSiO_2_-PFP-PEG
PET: Positron emission tomography
DCs: Dendritic cells
NIR: Near-infrared
ICG: Indocyanine green
NV: Nivolumab
ZIFs: Zeolitic imidazolate frameworks
TPZ: Tirapazamine
aAPCs: Artificial AP*Cs*
